# Phase II study of trifluridine/tipiracil (TAS-102) therapy in elderly patients with colorectal cancer (T-CORE1401): geriatric assessment tools and plasma drug concentrations as possible predictive biomarkers

**DOI:** 10.1007/s00280-021-04277-3

**Published:** 2021-05-24

**Authors:** Masanobu Takahashi, Yasuhiro Sakamoto, Hisatsugu Ohori, Yasushi Tsuji, Michio Kuroki, Satoshi Kato, Kazunori Otsuka, Keigo Komine, Masahiro Takahashi, Shin Takahashi, Hidekazu Shirota, Kota Ouchi, Yoshikazu Takahashi, Hiroo Imai, Hiroyuki Shibata, Takashi Yoshioka, Masaki Tanaka, Hiroaki Yamaguchi, Takuhiro Yamaguchi, Hideki Shimodaira, Chikashi Ishioka

**Affiliations:** 1 Department of Medical Oncology, Tohoku University Hospital, Seiryo-machi 1-1, Aoba-ku, Sendai, Miyagi 980-8574 Japan; 2 Department of Clinical Oncology, Institute of Development, Aging and Cancer, Tohoku University, Seiryo-machi 4-1, Aoba-ku, Sendai, Miyagi 980-8575 Japan; 3 Department of Medical Oncology, Osaki Citizen Hospital, 3-8-1 Honami, Furukawa, Osaki, Miyagi 989-6183 Japan; 4 Department of Clinical Oncology, Japanese Red Cross Ishinomaki Hospital, 71 Hebitanishimichishita, Ishinomaki, Miyagi 986-8522 Japan; 5 Department of Medical Oncology, Tonan Hospital, 7-3-8 kitashijonishi, Chuo-ku, Sapporo, Hokkaido 060-0004 Japan; 6 Department of Gastroenterology, Yamagata City Hospital Saiseikan, 1-3-26 Nanukamachi, Yamagata, Yamagata 990-8533 Japan; 7 Department of Medical Oncology, Iwate Prefectural Central Hospital, Ueda 1-1-1, Morioka, Iwate 020-0066 Japan; 8 Department of Medical Oncology, Miyagi Cancer Center, Nodayama 47-1, Medeshima, Natori 981-1293 Japan; 9 Department of Clinical Oncology, Graduate School of Medicine, Akita University, Hondo 1-1-1, Akita, 010-8543 Japan; 10 Department of Clinical Oncology, Yamagata University School of Medicine, Iida-nishi 2-2-2, Yamagata, 990-9585 Japan; 11 Department of Pharmaceutical Sciences, Tohoku University Hospital, Seiryo-machi 1-1, Aoba-ku, Sendai, Miyagi 980-8574 Japan; 12 Division of Biostatistics, Tohoku University Graduate School of Medicine, Seiryo-machi 2-1, Aoba-ku, Sendai, Miyagi 980-8575 Japan; 13 Department of Clinical Oncology, Tohoku University Graduate School of Medicine, Seiryo-machi 2-1, Aoba-ku, Sendai, Miyagi 980-8575 Japan

**Keywords:** Trifluridine, Tipiracil, Geriatric assessment, G8, Plasma concentration, Colorectal cancer

## Abstract

**Purpose:**

The current study aimed to determine the efficacy of trifluridine/tipiracil for elderly patients with advanced colorectal cancer.

**Methods:**

This single-arm, open-label, multicenter, phase II study included elderly patients aged 65 years or more who had fluoropyrimidine-refractory advanced colorectal cancer and received trifluridine/tipiracil (70 mg/m^2^, days 1–5 and 8–12, every 4 weeks). The primary endpoint was progression-free survival (PFS), while secondary endpoints included overall survival (OS), overall response rate (ORR), toxicities, association between efficacy and geriatric assessment scores, and association between toxicity and plasma drug concentrations.

**Results:**

A total of 30 patients with a mean age of 73 years were enrolled. Median PFS was 2.3 months (95% confidence interval, 1.9–4.3 months), while median OS was 5.7 months (95% confidence interval, 3.7–8.9 months). Patients had an ORR of 0%, with 57% having stable disease. Grade 4 neutropenia was observed in 13% of the patients. Patients with a higher G8 score (15 or more) showed longer PFS than those with a lower G8 score (median 4.6 vs. 2.0 months; *p* = 0.047). Moreover, patients with grade 3 or 4 neutropenia showed higher maximum trifluridine concentrations than those with grade 1 or 2 neutropenia (mean 2945 vs. 2107 ng/mL; *p* = 0.036).

**Discussion:**

The current phase II trial demonstrated that trifluridine/tipiracil was an effective and well-tolerated option for elderly patients with advanced colorectal cancer. Moreover, geriatric assessment tools and/or plasma drug concentration monitoring might be helpful in predicting the efficacy and toxicities in elderly patients receiving this drug.

**Trial registration number:**

UMIN000017589, 15/May/2015 (The University Hospital Medical Information Network)

**Supplementary Information:**

The online version contains supplementary material available at 10.1007/s00280-021-04277-3.

## Introduction

Recent notable progress in drug therapy has promoted longer survival in patients with metastatic or recurrent colorectal cancer. Cytotoxic drugs, such as fluoropyrimidines, irinotecan, and oxaliplatin, combined with molecularly targeted drugs, such as VEGF pathway inhibitors or anti-EGFR antibodies when the tumor *RAS* gene is wild-type, have been used in front-line therapy. Similarly, TAS-102 (trifluridine/tipiracil, FTD/TPI) and regorafenib have been widely utilized in salvage line therapy [1].

FTD/TPI is a nucleoside anti-tumor agent consisting of an active cytotoxic component, FTD, and a potent inhibitor of thymidine phosphorylase, TPI hydrochloride at a molar ratio of 1:0.5 [2]. A randomized phase II J003 trial showed that FTD/TPI exhibits promising efficacy and manageable toxicities in patients with colorectal cancer refractory to standard chemotherapy [2]. More recently, the phase III RECOURSE trial showed that FTD/TPI promoted better overall survival (OS) compared to placebo in patients with colorectal cancer refractory to standard chemotherapy, including fluoropyrimidines, irinotecan, and oxaliplatin [median OS 7.1 vs. 5.3 months; hazard ratio (HR) 0.68 95% confidence interval (CI) 0.58–0.81] [3]. Thus, FTD/TPI has become one of many options for third-line or later therapy in patients with metastatic colorectal cancer [4, 5].

Treatment strategies for elderly patients with colorectal cancer, particularly those with poor performance status (PS) or frailty, remain to be established. Subgroup analysis in a randomized phase II study suggested that FTD/TPI therapy similarly benefited both elderly patients aged 65 or older (HR 0.51, 95% CI 0.29–0.90) and younger patients (HR 0.64, 95% CI 0.39–1.03) [2]. However, majority of the patients enrolled in the randomized phase II study, except four patients, had a good Eastern Cooperative Oncology Group PS (0–1). In 2014, at the time when we planned this phase II trial, the phase III RECOURSE trial [3] had not been reported. Therefore, whether FTD/TPI is effective and tolerable for elderly patients with poor PS in daily clinical settings still remained unknown.

The International Society of Geriatric Oncology suggested that geriatric assessment (GA) may be useful in predicting cancer treatment-related toxicities and OS, as well as guiding treatment choice and intensity, by detecting impairments in elderly patients with cancer that are otherwise overlooked during routine physical examinations or medical history [6]. GA is a multidimensional and interdisciplinary evaluation tool that identifies functional, nutritional, cognitive, psychological, social support, and comorbidity factors [7, 8]. Although GA may help guide treatment decisions in oncology, a full GA is time-consuming. Nonetheless, geriatric screening tools, such as G8, Vulnerable Elders Survey-13, and the Flemish version of the Triage Risk Screening Tool (fTRST), have been recommended for identifying patients needing further evaluation with a full GA [9, 10]. Our previous retrospective analysis had shown that a lower G8 score was associated with worse survival in patients aged 70 years or more with various advanced cancers, mainly including gastrointestinal cancer. Moreover, the same study found that the combination of G8 scores and PS had better prognostic value than PS alone, with such a combination having been widely accepted as a reliable prognostic marker for patients with cancer [11]. However, whether G8 scores can predict clinical outcomes in patients with advanced cancer from chemotherapy remains to be elucidated.

The present phase II study aimed to evaluate the efficacy and toxicity of FTD/TPI in elderly patients with colorectal cancer and elucidate whether plasma FTD/TPI concentrations and GA screening score are associated with clinical outcomes in patients receiving FTD/TPI.

## Methods

### Patients and study design

This single-arm, phase II trial enrolled patients from eight institutions affiliated with the Tohoku Clinical Oncology Research and Education Society (T-CORE) in Japan. The study was conducted in accordance with the Declaration of Helsinki, and the study protocol was approved by institutional ethics committees and/or institutional review board of all participating sites. Written informed consent was obtained from all patients prior to inclusion.

Patients aged 65–85 years; had histologically confirmed unresectable or metastatic colorectal cancer; had PS of 0–2; were able to take medication orally; received one or more chemotherapeutic regimen(s), including fluoropyrimidines; projected to survive at least 3 months after study enrollment; had appropriate bone marrow and liver functions (WBC ≥ 3000/mm^3^, neutrophil ≥ 1500/mm^3^, hemoglobin ≥ 8.0 g/dL, platelet ≥ 10.0 × 10^4^/mm^3^, aspartate aminotransferase ≤ 100 U/L, alanine aminotransferase ≤ 100 U/L, total bilirubin < 2.0 mg/dL) were deemed eligible for inclusion.

### Treatment

FTD/TPI was administered a dose of 35 mg/m^2^ twice daily for days 1–5 and days 8–12, every 4 weeks.

### Endpoints

The primary endpoint was progression-free survival (PFS), while secondary endpoints included OS, time to treatment failure (TTF), overall response rate (ORR), toxicities, relationship between GA and effectiveness/toxicities, relationship between plasma FTD concentrations and GA, and relationship between plasma FTD concentration and effectiveness/toxicities.

PFS was defined as the duration from treatment protocol initiation to the first radiologic confirmation of disease progression or death from any cause. OS was defined as the duration from treatment protocol initiation to death from any cause. TTF was defined as the duration from treatment protocol initiation to its cessation from any cause. Tumor response was evaluated through computed tomography every 4 weeks within the first 2 months and then every 8 weeks thereafter, according to the Response Evaluation Criteria in Solid Tumors version 1.1. ORR was defined as the number of patients with a complete response (CR) or partial response (PR) divided by the total number of patients with measurable lesions. The disease control rate (DCR) was defined as the number of patients with CR, PR, or stable disease (SD) divided by the number of patients in whom response could be evaluated.

### Geriatric assessments

All patients enrolled herein underwent GA by physicians using the G8 screening tool and fTRST, as described previously [11, 12].

### Measurements of FTD and TPI

The plasma concentrations of FTD and TPI were measured using a LCMS-8050 triple quadrupole mass spectrometer coupled with a Nexera X2 UHPLC system (Shimadzu, Kyoto, Japan). Fifty-microliters of plasma were mixed with 50 μL of 10 μg/mL 5-chlorouracil (internal standard) dissolved in acetonitrile and then 100 μL of methanol. The mixture was vortexed and centrifuged at 15,000×*g* for 5 min. To prepare a sample for injection, 200 μL of water was added to 100 μL of the supernatant.

For FTD analysis, chromatographic separation was achieved using a Shim-pack GIS C18 column (75 × 2.1 mm i.d., 3 μm, Shimadzu), which was maintained at 40 °C. The mobile phase consisted of solution A (10 mM ammonium acetate in water) and solution B (methanol), which formed the following gradient: 15% B (0–1.75 min); 15–80% B (1.75–3 min); 80% B (3–5 min); and 15% B (5–7 min). The flow rate of the mobile phase was 0.3 mL/min. The LCMS-8050 was equipped with an electrospray ionization source operating in positive and negative ion detection mode. During selected reaction monitoring, the *m*/*z* transitions 319.00 → 203.00 and 145.05 → 42.05 monitored FTD and 5-chlorouracil, respectively. Injection volume of sample was 5 μL.

For TPI analysis, chromatographic separation was achieved using a Luna HILIC column (50 × 2 mm i.d., 3 μm, Phenomenex, Torrance, CA, USA), which was maintained at 40 °C. The mobile phase consisted of solution A (10 mM ammonium acetate in water) and solution B (acetonitrile), which formed the following gradient: 90% B (0–1 min); 90–50% B (1–2 min); 50% B (2–5 min); and 90% B (5–7 min). The flow rate of the mobile phase was 0.3 mL/min. The LCMS-8050 was equipped with an electrospray ionization source operating in positive and negative ion detection mode. During selected reaction monitoring, the *m*/*z* transitions 243.25 → 183.20 and 145.05 → 42.05 for TPI and 5-chlorouracil, respectively. Injection volume of sample was 1 μL.

The calibration curves for FTD and TPI were linear in the range 10–2000 ng/mL. Linearity was achieved with a correlation coefficient (R2) > 0.995.

### Pharmacokinetics

Plasma concentrations of FTD and TPI were monitored at five time points on day 8, including before, 45 min, 90 min, 180 min, and 360 min after FTD/TPI administration in the morning. The area under curve (AUC) of the plasma concentration from 0 to 10 h of FTD and TPI [13] were analyzed by a noncompartmental model from the plasma concentration of each blood sampling time (Phoenix WinNonlin software Version 7.0, Certara USA, Inc., Princeton, NJ, USA).

### Statistical analysis

This study was designed to have 90% power to detect a threshold value of 1.0 months and an expected of 2.0 months for median PFS, based on the previous phase II data [2] throughout the 2-year registration period and 1-year follow-up period, with a two-sided type I error rate of 0.025. The minimum number of patients required was estimated to be 22. To account for attrition, a total of 30 patients were planned to be enrolled in this study.

## Results

### Patient characteristics

A total of 30 patients (median age 73 years; range 65–81 years; 21 men and 9 women) were enrolled in this phase II study conducted between August, 2015 and June, 2016. The median follow-up period was 5.7 months. The clinicopathological characteristics of patients included herein are summarized in Table 1. Among the included patients, 21 had colon cancer, while 9 had rectal cancer. All patients received two or more previous chemotherapeutic regimens (median 3).

**Table 1:** Characteristics of patients enrolled in this study (*N* = 30)

Factor	*N*	%
Sex		
Men	21	70.0
Women	9	30.0
Age		
Median	73	
Range	65–81	
Primary site		
Colon	21	70.0
Rectum	9	30.0
Recurrent or metastatic site		
Liver	20	66.7
Lung	13	43.6
LN	6	20.0
Peritoneum	2	6.7
Local	2	6.7
Histology-differentiation of adenocarcinoma		
Well	3	10.0
Moderately	14	46.7
Poorly	10	33.3
Unknown	3	10.0
History of surgery		
Yes	23	76.7
Previous chemotherapeutic regimens		
2	13	43.3
3	7	23.3
4 or more	10	33.3
Median	3	
PS		
0	11	36.7
1	16	53.3
2	3	10.0
G8		
15–17	8	26.7
0–14	22	73.3
fTRST		
0–1	18	60.0
2–5	12	40.0

*PS* ECOG-performance status, *fTRST* the Flemish version of the triage risk screening tool

### Efficacy

**Fig. 1:**
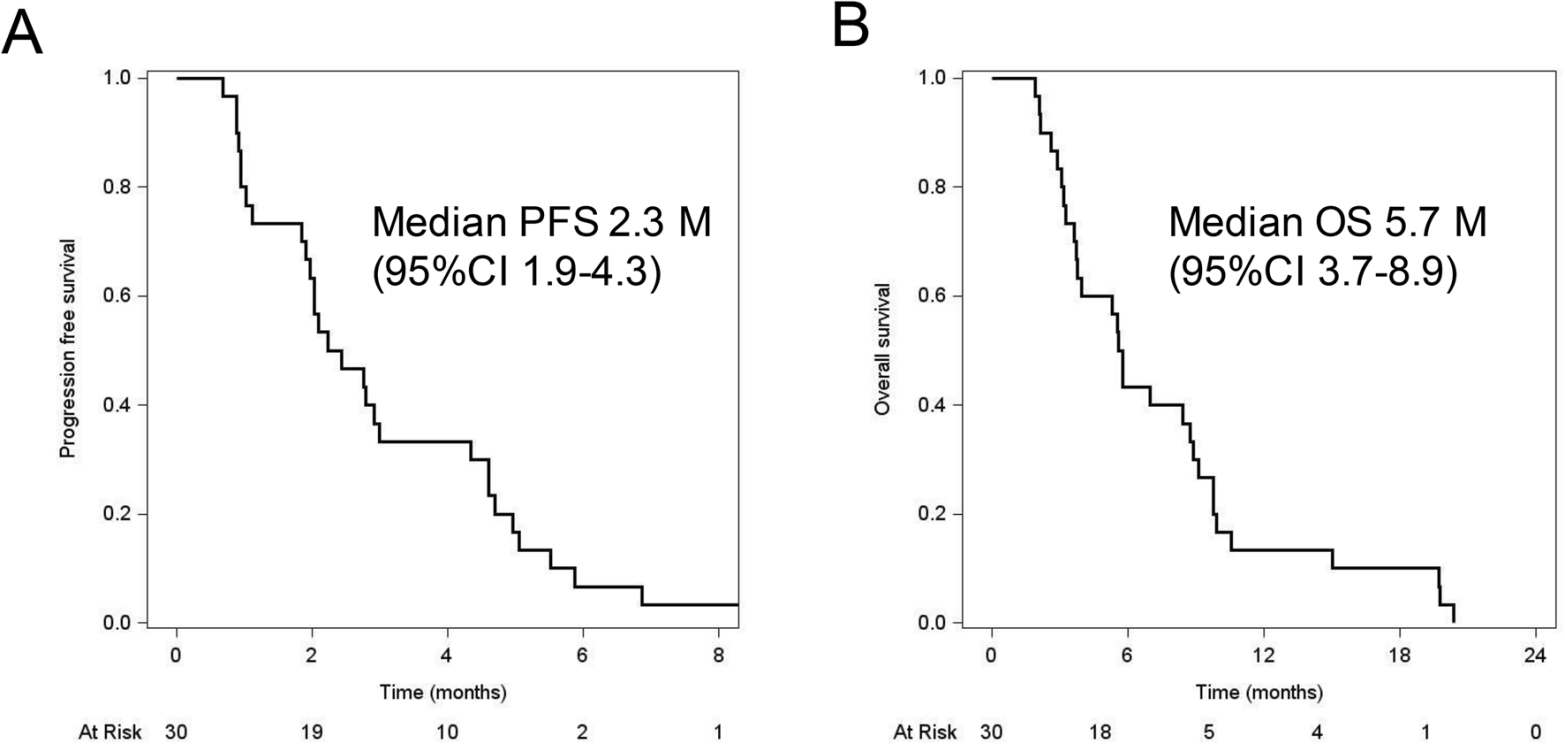
Kaplan–Meyer curve for **a** progression-free survival and **b** overall survival of patients enrolled in this study

Patients had a median PFS of 2.3 months (95% CI 1.9–4.3; Fig. 1a). This study met its primary end point, predefined as PFS with the lower limit of the 95% CI being > 1.0 months. Median OS was 5.7 months (95%CI 3.7–8.9; Fig. 1b). Among evaluable patients, ORR was 0%, while DCR was 61% (17/28).

### Adverse events

Treatment-related adverse events were observed in all 30 patients (100%). Commonly observed adverse events (> 40%) included anemia (100%), neutropenia (83%), and thrombocytopenia (57%), anorexia (47%), and fatigue (43%), as shown in Table 2. Grade 3 or 4 adverse events were observed in 80% of the patients, with the most commonly observed ones (> 10%) being neutropenia (47%), anemia (17%), and anorexia (13%). Febrile neutropenia, as well as treatment-related death, was not observed.

**Table 2:** Adverse events observed in patients enrolled in this study (*N* = 30)

Factor	Grade *N* (%)					
	1	2	3	4	All	3–4
Hematological						
Neutropenia	5 (16.7)	6 (20.0)	10 (33.3)	4 (13.3)	25 (83.3)	14 (46.7)
Anemia	9 (30.0)	16 (53.3)	4 (13.3)	1 (3.3)	30 (100.0)	5 (16.7)
Thrombocytopenia	16 (53.3)	0 (0.0)	1 (3.3)	0 (0.0)	17 (56.7)	1 (3.3)
Non-hematological						
Nausea	6 (20.0)	5 (16.7)	0 (0.0)	0 (0.0)	11 (36.7)	0 (0.0)
Vomiting	3 (10.0)	0 (0.0)	0 (0.0)	0 (0.0)	3 (10.0)	0 (0.0)
Anorexia	6 (20.0)	4 (13.3)	4 (13.3)	0 (0.0)	14 (46.7)	4 (13.3)
Fatigue	7 (23.3)	6 (20.0)	0 (0.0)	0 (0.0)	13 (36.7)	0 (0.0)
Fever up	3 (10.0)	1 (3.3)	0 (0.0)	0 (0.0)	4 (13.3)	0 (0.0)
Abdominal pain	1 (3.3)	0 (0.0)	1 (3.3)	0 (0.0)	2 (6.7)	1 (3.3)
Diarrehea	6 (20.0)	1 (3.3)	0 (0.0)	0 (0.0)	7 (23.3)	0 (0.0)
Stomatitis	1 (3.3)	2 (6.7)	0 (0.0)	0 (0.0)	3 (10.0)	0 (0.0)
Palpitation	1 (3.3)	0 (0.0)	0 (0.0)	0 (0.0)	1 (3.3)	0 (0.0)

### G8 and fTRST

A total of eight patients showed favorable, higher G8 scores (15–17), whereas 22 showed unfavorable, lower scores (14 or less). Moreover, 18 patients showed favorable, lower fTRST scores (0–1), whereas 12 showed unfavorable, higher scores (2–5).

**Fig. 2:**
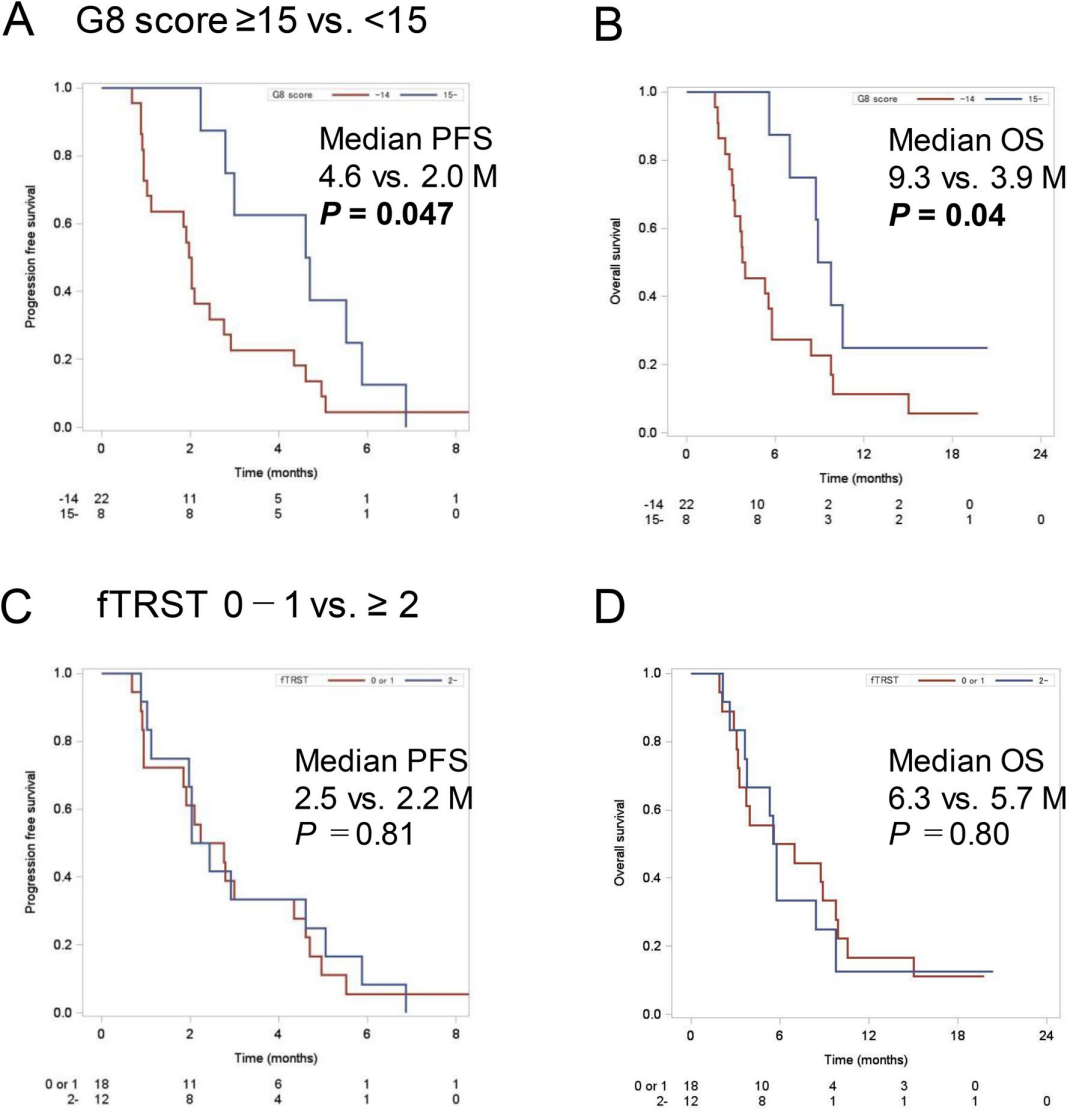
**a** Progression-free survival (PFS) and **b** overall survival (OS) of patients enrolled in this study according to G8 score (15 or more vs. 14 or less). **c** PFS and **d** OS of patients enrolled in this study according to the Flemish version of the Triage Risk Screening Tool score (0–1 vs. 2 or more)

Patients with higher G8 scores (15–17) showed longer PFS (median 4.6 vs. 2.0 months; *p* = 0.047 using the logrank test; Fig. 2a) and OS (median 9.3 vs. 3.9 months; *p* = 0.04 using the logrank test; Fig. 2b) than those with lower G8 scores. Moreover, patients with higher G8 scores (15–17) showed higher DCR than those with lower G8 scores (100% vs. 45%; *p* = 0.01 using Fisher’s exact test). Furthermore, when the G8 cutoff value was set to 14, higher G8 scores (14–17) were even more significantly associated with favorable PFS (*p* = 0.008 using the logrank test) and OS (*p* = 0.0028; Supplementary Fig. 1). However, patients with low fTRST scores did not exhibit longer PFS (median 2.5 vs. 2.2 months; *p* = 0.81 using the logrank test; Fig. 2c) or OS (median 6.3 vs. 5.7 months; *p* = 0.81 using the logrank test; Fig. 2d) compared to those with high fTRST scores. Likewise, patients with low fTRST scores did not have higher DCR (73% vs. 53%; *p* = 0.43 using Fisher’s exact test) compared to those with high fTRST scores.

Patients with good PS (0) did not exhibit significantly longer PFS (median 3.0 vs. 2.2 months; *p* = 0.55 using the logrank test; Supplementary Fig. 2A) or OS (median 8.8 vs. 5.3 months; *p* = 0.21 using the logrank test; Supplementary Fig. 2B) compared to those with worse PS (1–2).

### Plasma FTD and TPI concentrations

**Fig. 3:**
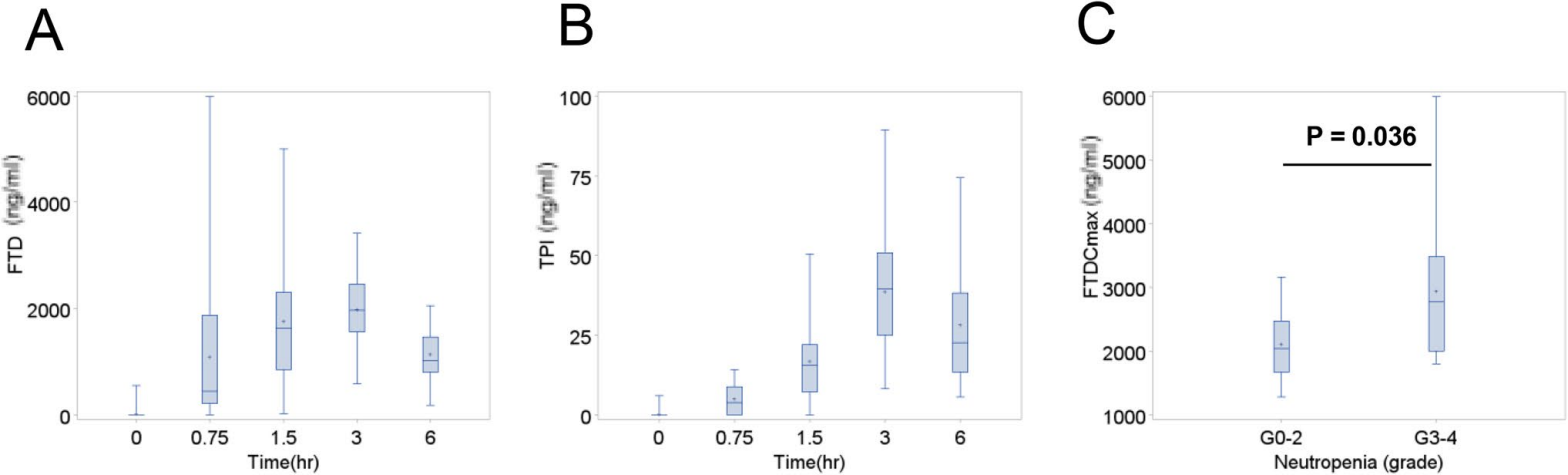
Time-dependent changes in plasma concentrations of **a** trifluridine (FTD) and **b** tipiracil (TPI) before, 45 min, 1.5 h, 3 h, and 6 h after oral trifluridine administration on day 8 of the first course. Maximum concentration (Cmax) of FTD according to **c** grade 0–2 vs. grade 3–4

Plasma concentrations of FTD and TPI were available in 25 patients and were highest 3 h after administration (mean ± standard deviation: FTD 1979 ± 771 ng/mL, TPI 38.7 ± 20.7 ng/mL; Fig. 3a, b). FTD had a maximum concentration (Cmax) of 2509 ± 1015 ng/mL and an area under the curve (AUC) of 12,500 ± 4728 ng·h/mL (Table 3). Meanwhile, TPI had a Cmax of 43.6 ± 20.4 ng/mL and an AUC of 221 ± 112 ng·h/mL (Table 3).

**Table 3:** Pharmacokinetics of trifluridine/tipiracil

Factor	Mean	SD
FTD		
AUC ng h/mL	12,500.6	4727.5
Cmax ng/mL	2509.3	1015.3
TPI		
AUC ng h/mL	221.3	112.1
Cmax ng/mL	43.6	20.4

*FTD* trifluridine, *TPI* tipiracil

Given that neutropenia was the most frequently observed adverse event in patients who received FTD/TPI, we attempted to further elucidate whether plasma FTD and/or TPI concentrations were associated with frequency of neutropenia occurrence. Accordingly, patients with grade 3 or 4 neutropenia (*N* = 12) showed a higher FTD Cmax than those with grade 1 or 2 neutropenia (mean 2945 vs. 2107 ng/mL; *p* = 0.036 using Student’s *t* test; Fig. 3c). However, the FTD AUC was not statistically significantly associated with neutropenia (data not shown). Meanwhile, neither TPI Cmax nor AUC was associated neutropenia (data not shown).

We subsequently determined whether a correlation existed between FTD Cmax and efficacy. After classifying patients into the high (*N* = 13) and low (*N* = 12) FTD Cmax group using the median value as the cutoff, no significant difference in either PFS or OS was observed between both two groups (data not shown). Conversely, the high Cmax group more frequently exhibited SD compared to the low Cmax group (85% vs. 33%; *p* = 0.015 using Fisher’s exact test).

## Discussion

The current phase II trial demonstrated that FTD/TPI can be an effective and well-tolerated option in elderly patients with advanced colorectal cancer considering that all patients met the primary end point. Moreover, GA screening tools and/or plasma drug concentration monitoring might be helpful for predicting the efficacy and toxicities in elderly patients receiving this drug.

The present study obtained a median PFS 2.3 months (95% CI 1.9–4.3 months), a median OS of 5.7 months (95% CI 3.7–8.9 months), an ORR of 0%, and DCR of 61%, respectively. In their randomized phase II J003 study, Yoshino et al. found a median PFS and OS of 2.0 months (95% CI 1.9–2.8) and 9·0 months (95% CI 7.3–11.3) in the FTD/TPI arm and 1.0 months (95% CI 1.0–1.0) and 6·6 months (95% CI 4.9–8.0) in the placebo group (HR 0.41, 95% CI 0.28–0.59; HR 0.56, 95% CI 0.39–0.81), respectively [2]. ORR and DCR were 1% and 44% [2]. More recently, after having started the present study, the RECOURSE study obtained a median PFS and OS of 2.0 and 7.1 months in the FTD/TPI arm and 1.7 months (HR 0.48, 95% CI 0.41–0.57) and 5.3 months (HR 0.68, 95% CI 0.58–0.81) in the placebo arm, respectively [3]. ORR and DCR were achieved in 1.6% and 44% of the FTD/TPI arm [3]. Although our phase II study included only elderly patients aged 65 years or older, no apparent difference in efficacy was observed between the our study and the aforementioned studies. Subgroup analysis in the RECOURSE study also suggested that patients aged 65 years or older similarly benefited from FTD/TPI therapy (PFS: HR 0.41, 95% CI 0.32–0.52; OS: HR 0.62, 95% CI 0.48–0.80) compared to those aged less than 65 years (PFS: HR 0.52, 95% CI 0.42–0.65; OS, HR 0.74, 95% CI 0.59–0.94) [3]. The efficacy of FTD/TPI observed in the current study seems to be consistent with that presented in the RECOURSE study. Moreover, considering that our study also included patients with a PS of 2, albeit relatively small in number (*N* = 3), FTD/TPI treatment in elderly patients in our study can be considered efficient enough.

Treatment tolerability observed herein seemed to be similar to that reported in previous studies. Although all patients included in our study exhibited adverse events, most of them were hematological with no severe cases. Grade 3–4 toxicities, such as neutropenia (46.7%), anemia (16.7%), and anorexia (13.3%) had been observed in 80% of our patients. However, the aforementioned toxicities were mostly manageable, while treatment-related death was not observed. On the other hand, the RECOURSE trial showed that 98% and 69% of patients exhibited any event and grade 3 or higher events, with one treatment-related death, in FTD/TPI arm [3].

One of the clinically important points revealed in the present study was that G8 scoring, a screening tool for GA, might be helpful for predicting the efficacy of FTD/TPI in elderly patients with colorectal cancer. A recent systematic review showed that 15 of the 24 studies screened, including our previous studies, found an association between G8 score and survival [11, 14]. However, only a handful of reports have analyzed G8 score as a predictive biomarker for a specific chemotherapy in a specific type of cancer, with their results being controversial. One of the studies including a large number of patients with a specific type of cancer showed that among their cohort of 252 patients with colorectal cancer who received chemotherapy ± bevacizumab, G8 scores were significantly associated with PFS (11.4 months with a score of more than 14 vs. 8.7 months with a score of 14 or less; *p* = 0.021) during univariate analysis, although PS was more significantly associated with PFS (PFS of 4.8 months with a PS of two or more vs. PFS of 8.8 months with PS of 1 and 10.3 months with a PS of 2; *p* < 0.0001) [15]. In contrast, subgroup analysis from the PRODIGE 20 randomized phase II trial showed that no geriatric variable, including G8 score, predicted PFS or OS in 102 patients with colorectal cancer aged 75 years or older who received chemotherapy ± bevacizumab [16]. Although the current study found that G8 score was significantly associated with PFS and OS in elderly patients with colorectal cancer who received FTD/TPI, such findings should be further validated in future studies.

Accumulating reports have suggested the association between neutropenia onset and favorable clinical outcomes among patients receiving FTD/TPI [17, 18]. Such results support the notion that maintained blood concentrations of FTD/TPI contribute to both efficacy and toxicity in patients. Indeed, a phase I study proposed a correlation between drug plasma concentrations and toxicity after determining that FTD Cmax and AUC monitored on day 12 of the first course (AUC_0-10 h_) were significantly inversely correlated with neutrophil count [13]. More recently, Yoshino et al. have revealed associations between plasma FTD concentrations and toxicities/efficacies using larger data [19]. Accordingly, their post hoc analysis using subset data from the J003 trial [2] and RECOURSE trial [3] revealed that patients with high FTD AUC (above median *N* = 69), monitored on day 12 of the first course, had significantly increased neutropenia (any grade 84% vs. 59%; grade 3 or more, 48% vs. 30%) compared to those with low FTD AUC (*N* = 69) [19]. Moreover, the high FTD AUC group tended to have longer OS compared to the low FTD group, although the difference was not statistically significant (median 9.2 vs. 7.2 months, HR 0.72, 95% CI 0.46–1.11). PFS did not differ significantly between both groups (HR 0.82, 95% CI 0.57–1.18). The current study showed that FTD Cmax on day 8 of the first course was significantly associated with the onset of G3–4 neutropenia (Fig. 3c) and DCR. Those findings are consistent with previous reports [13, 19], in spite of some differences in sampling timing, AUC, and Cmax. Cmax of FTD in our study (2509 ± 1015 ng/mL in day 8) seems lower than that in day 12 in the Doi’s study (4752 ± 1697 ng/mL in day 12) [13] and in the Yoshino’s study (median 5000 ng/mL in day 12) [19], but seems comparable to that in day 1 in the Doi’s study (3338 ± 767 ng/mL in day 1). Based on that Cmax of FTD should be higher in day 12 than day 1 or day 8 as FTD/TPI is administered on days 1–5 and 8–12, those results between the previous other studies and ours seem to some extent consistent. In addition, AUC_0-10 h_ of FTD in our study (12,500 ± 4727 ng·h/mL in day 8) seemed higher than that in day 1 in the Doi’s study (8678 ± 1786 ng·h/mL) and lower than in day 12 in the Doi’s study (20,950 ± 2237 ng·h/mL) and in the Yoshino’s study, where the information on the detailed calculation was not available (median 43,510 ng·h/mL) [19]. The AUC results of the previous studies and our studies seem also consistent. Nevertheless, the parameter (AUC, Cmax, or others) or timing related to FTD plasma concentrations that best predicts efficacy and toxicity of FTD/TPI and that is most clinically useful remains to be elucidated.

Our study has several limitations worth noting. First, this was a single-arm study. Second, a relatively small sample size had been included herein. Nevertheless, the primary endpoint was achieved, with subgroup analysis suggesting that G8 and plasma drug concentrations could be promising predictive biomarkers in elderly patients with colorectal cancer, warranting further validation in future studies.

In conclusion, the present phase II study, although a limitation of a single-arm setting should be noted, suggested that FTD/TPI can be a sufficiently effective and tolerable option in elderly patients with advanced colorectal cancer. Moreover, plasma FTD concentrations, and particularly GA screening tools, such as G8, could serve as clinically useful predictive biomarkers for efficacy and toxicity in the management of elderly patients with colorectal cancer receiving FTD/TPI therapy.

## Supplementary Information

Below is the link to the electronic supplementary material.

Supplementary file1 (PPTX 147 KB) Supplementary Figure 1 (A) Progression-free survival and (B) overall survival of patients enrolled in this study according to G8 score (14 or more vs. 13 or less). Supplementary Figure 2 (A) Progression-free survival and (B) overall survival of patients enrolled in this study according to Eastern Cooperative Oncology Group Performance Status (0 vs. 1–2).

## Data Availability

The data analyzed in this study are available from the corresponding author upon request.
