# Supplementary material for: Phase II study of trifluridine/tipiracil (TAS-102) therapy in elderly patients with colorectal cancer (T-CORE1401): geriatric assessment tools and plasma drug concentrations as possible predictive biomarkers
Source: Cancer Chemother Pharmacol. 2021 May 24;88(3):393–402. doi: 10.1007/s00280-021-04277-3 (PMC8316169; doi:10.1007/s00280-021-04277-3)
Supplement: Supplementary file 1 — Supplementary file1 (PPTX 147 KB) Supplementary Figure 1 (A) Progression-free survival and (B) overall survival of patients enrolled in this study according to G8 score (14 or more vs. 13 or less). Supplementary Figure 2 (A) Progression-free survival and (B) overall survival of patients enrolled in this study according to Eastern Cooperative Oncology Group Performance Status (0 vs. 1–2). [file 280_2021_4277_MOESM1_ESM.pptx]

## Slide 1
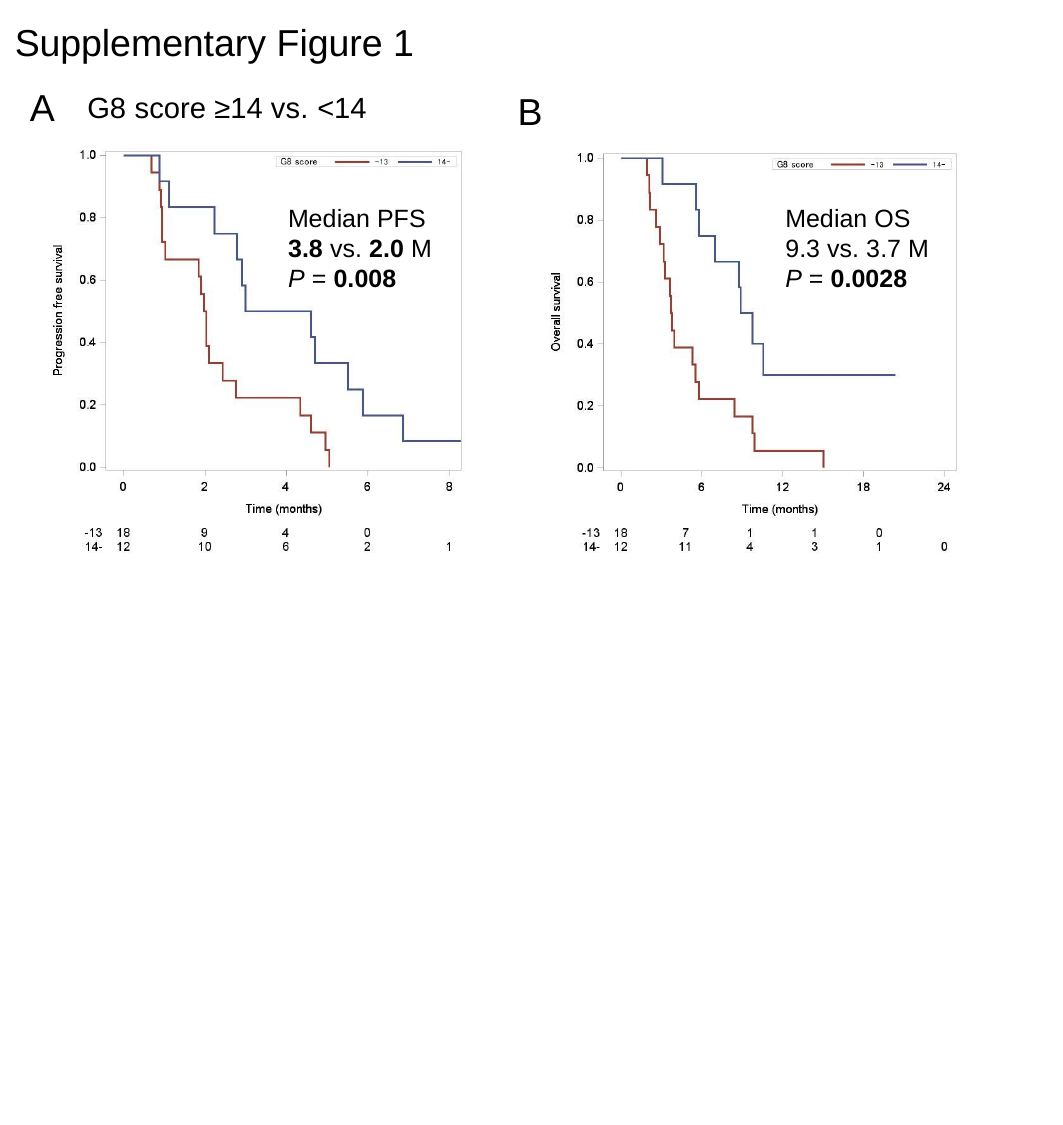

Supplementary Figure 1
A
B
G8 score ≥14 vs. <14
Median PFS
3.8 vs. 2.0 M
P = 0.008
Median OS
9.3 vs. 3.7 M
P = 0.0028

## Slide 2
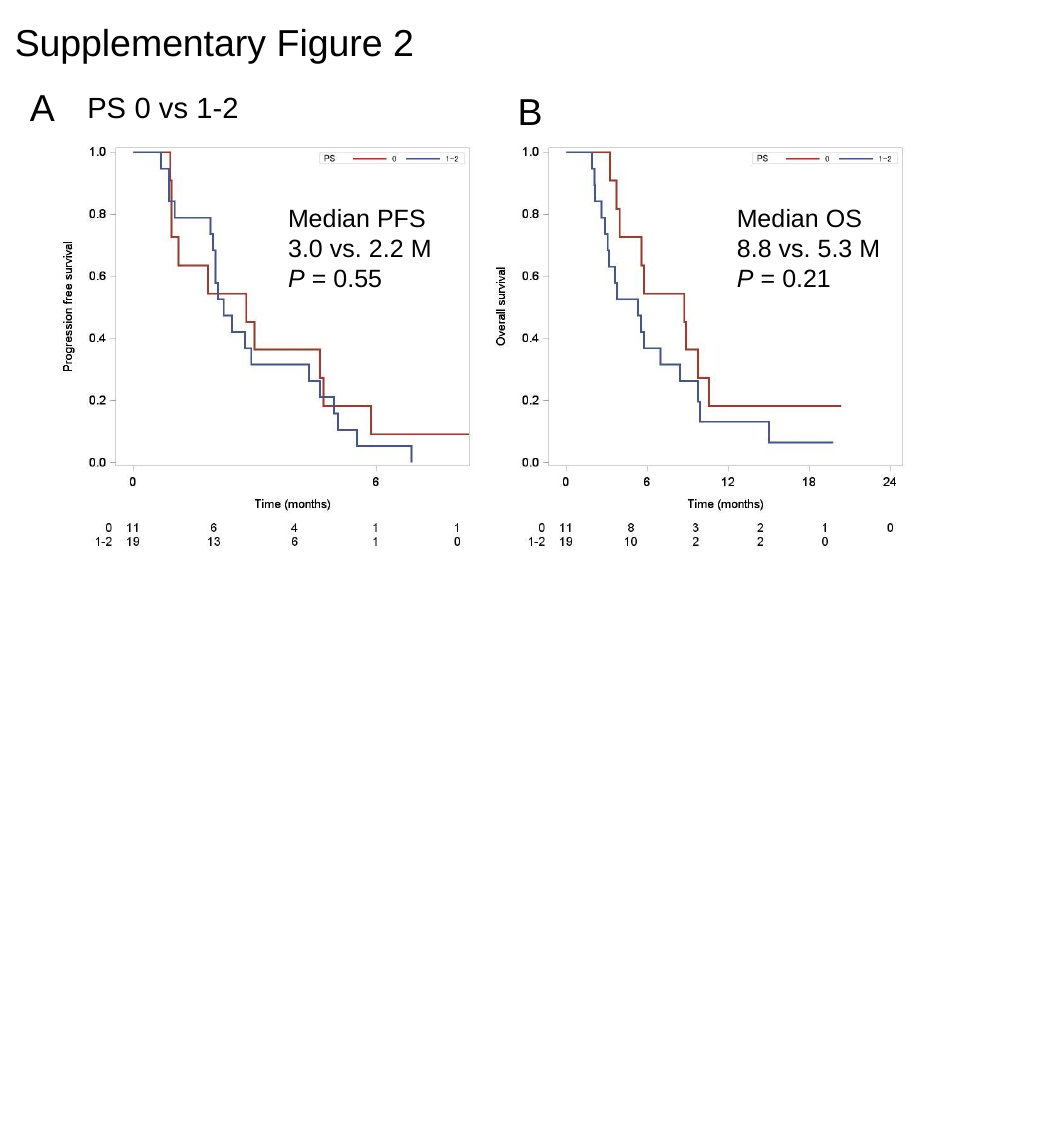

Supplementary Figure 2
A
B
PS 0 vs 1-2
Median PFS
3.0 vs. 2.2 M
P = 0.55
Median OS
8.8 vs. 5.3 M
P = 0.21
